# Association of C-Reactive Protein Velocity with Early Left Ventricular Dysfunction in Patients with First ST-Elevation Myocardial Infarction

**DOI:** 10.3390/jcm10235494

**Published:** 2021-11-24

**Authors:** Magdalena Holzknecht, Christina Tiller, Martin Reindl, Ivan Lechner, Priscilla Fink, Patrick Lunger, Agnes Mayr, Benjamin Henninger, Christoph Brenner, Gert Klug, Axel Bauer, Bernhard Metzler, Sebastian Johannes Reinstadler

**Affiliations:** 1 University Clinic of Internal Medicine III Cardiology and Angiology, Medical University of Innsbruck, Anichstrasse 35, 6020 Innsbruck, Austria; Magdalena.Holzknecht@tirol-kliniken.at (M.H.); Christina.Tiller@tirol-kliniken.at (C.T.); Martin.Reindl@tirol-kliniken.at (M.R.); Ivan.Lechner@tirol-kliniken.at (I.L.); Priscilla.Fink@tirol-kliniken.at (P.F.); Patrick.Lunger@student.i-med.ac.at (P.L.); Christoph.Brenner@tirol-kliniken.at (C.B.); Gert.Klug@tirol-kliniken.at (G.K.); Axel.Bauer@tirol-kliniken.at (A.B.); Bernhard.Metzler@tirol-kliniken.at (B.M.); 2 University Clinic of Radiology, Medical University of Innsbruck, Anichstrasse 35, 6020 Innsbruck, Austria; A.Mayr@i-med.ac.at (A.M.); Benjamin.Henninger@tirol-kliniken.at (B.H.)

**Keywords:** ST-elevation myocardial infarction, C-reactive protein, left ventricular function, cardiac magnetic resonance imaging

## Abstract

C-reactive protein velocity (CRPv) has been proposed as a very early and sensitive risk predictor in patients with ST-elevation myocardial infarction (STEMI). However, the association of CRPv with early left ventricular (LV) dysfunction after STEMI is unknown. The aim of this study was to investigate the relationship between CRPv and early LV dysfunction, either before or at hospital discharge, in patients with first STEMI. This analysis evaluated 432 STEMI patients that were included in the prospective MARINA-STEMI (Magnetic Resonance Imaging In Acute ST-elevation Myocardial Infarction. ClinicalTrials.gov Identifier: NCT04113356) cohort study. The difference of CRP 24 ± 8 h and CRP at hospital admission divided by the time (in h) that elapsed during the two examinations was defined as CRPv. Cardiac magnetic resonance (CMR) imaging was conducted at a median of 3 (IQR 2–4) days after primary percutaneous coronary intervention (PCI) for the determination of LV function and myocardial infarct characteristics. The association of CRPv with the CMR-derived LV ejection fraction (LVEF) was investigated. The median CRPv was 0.42 (IQR 0.21–0.76) mg/l/h and was correlated with LVEF (r_S_ = −0.397, *p* < 0.001). In multivariable linear as well as binary logistic regression analysis (adjustment for biomarkers and clinical and angiographical parameters), CRPv was independently associated with LVEF (β: 0.161, *p* = 0.004) and LVEF ≤ 40% (OR: 1.71, 95% CI: 1.19–2.45; *p* = 0.004), respectively. The combined predictive value of peak cardiac troponin T (cTnT) and CRPv for LVEF ≤ 40% (AUC: 0.81, 95% CI 0.77–0.85, *p* < 0.001) was higher than it was for peak cTnT alone (AUC difference: 0.04, *p* = 0.009). CRPv was independently associated with early LV dysfunction, as measured by the CMR-determined LVEF, revealing an additive predictive value over cTnT after acute STEMI treated with primary PCI.

## 1. Introduction

Despite significant progress in the management of ST-elevation myocardial infarction (STEMI), left ventricular (LV) systolic dysfunction is the most common consequence after STEMI and has significant implications on short- and long-term prognosis [1,2,3]. Early knowledge of the individual risk of reduced ejection fraction post-STEMI is therefore desirable [4].

Elevated peak C-reactive protein (CRP) levels are associated with reduced LV ejection fraction (LVEF) [5], more severe myocardial tissue injury [6,7,8], and worse outcome in the setting of acute myocardial infarction [9,10,11]. However, peak CRP values are reached 2–3 days after acute STEMI, decelerating early risk stratification [6,12]. An association between CRP level dynamics and adverse cardiovascular events and death after acute coronary syndromes has been suggested [13]. According to Świątkiewicz et al., changes in CRP concentrations during STEMI might serve as a risk marker for post-infarct LV systolic dysfunction and heart failure [14,15,16], even years after the index event, as well as LV remodeling [17], underlining the clinical usefulness of CRP dynamics in this patient setting. In the CAMI-1 study, the CRP gradient was suggested to correlate with a greater extent of myocardial infarct size (IS) and reduced LVEF [18].

CRP velocity (CRPv), which displays CRP level changes over time, has been suggested as a very early and more sensitive parameter for more serious outcomes following STEMI [19,20,21,22]. However, the association of CRPv with LV systolic dysfunction has not been specifically investigated so far. The aim of this study was, therefore, to investigate the relationship between CRPv and LVEF, assessed by cardiac magnetic resonance (CMR) imaging, in patients with acute STEMI treated with primary percutaneous coronary intervention (PCI). We hypothesized that CRPv could predict LV dysfunction with a comparable accuracy to peak CRP and peak cardiac troponin T (cTnT) as reference standard biomarkers in this setting.

## 2. Methods

### 2.1. Study Design, Patient Population and Endpoint Definition

This study is based on the “Magnetic Resonance Imaging In Acute ST-Elevation Myocardial Infarction (MARINA-STEMI)” trial (ClinicalTrials.gov Identifier: NCT04113356), a prospective observational study recruiting acute STEMI patients, that were treated with primary PCI, at the coronary care unit of the Medical University of Innsbruck. The following inclusion criteria were applied for the present analysis: first STEMI according to the European Society of Cardiology/American College of Cardiology committee criteria [23], revascularization by primary PCI within 12 h after the onset of ischemic signs or symptoms, and Killip class < 3 at time of CMR imaging. The exclusion criteria were as follows: inability or unwillingness to sign written informed consent, age < 18 years, any history of a previous myocardial infarction or coronary intervention, high-sensitivity (hs) CRP > 15 mg/L at the time of hospital admission, fever (temperature > 38 °C) or having experienced an acute infection with fever within 14 days prior to study inclusion, chronic inflammatory disease, an estimated glomerular filtration rate < 30 mL/min per 1.73 m^2^, and any other contraindication to CMR examination (pacemaker, severe claustrophobia, orbital foreign body, cerebral aneurysm clip, or known or suggested contrast agent allergy to gadolinium) [19].

For the determination of hs-cTnT and hs-CRP, peripheral venous blood samples were performed and analyzed as described previously [24]. In brief, concentrations of CRP were measured on the cobas^®^ 8000 modular analyzer (Roche Diagnostics^®^), and cTnT measurements were conducted by applying a validated enzyme immunoassay (hs-cTnT; E170, Roche Diagnostics^®^). CRP and cTnT levels were assessed at hospital admission, 6 ± 2 h, 12 ± 4 h, 24 ± 8 h, and then daily until day 4 after PCI or discharge [25]. The difference between CRP 24 ± 8 h and CRP at hospital admission, divided by the time (in h) that elapsed during the two examinations, was defined as CRPv [19,21].

The primary objective of the current study was the association between CRPv and LVEF as determined by CMR imaging. The secondary objective was to assess the potential additive value of CRPv over cTnT for the prediction of LV dysfunction. The value of LVEF categorization ≤40% to define LV dysfunction is derived from the latest guidelines [26] and is based on previous analyses investigating the prognostic impact of reduced LVEF at any time after STEMI [27].

Prior to study inclusion, all participants gave written informed consent. The study was designed and conducted in accordance with the Declaration of Helsinki and received approval by the research ethics committee of the Medical University of Innsbruck.

### 2.2. Cardiac Magnetic Resonance Imaging

CMR examinations were performed in the supine position on a 1.5 Tesla clinical MR scanner (MAGNETOM Avanto fit; Siemens Healthineers AG, Erlangen, Germany) within the first week after treatment with primary PCI. The detailed standardized imaging protocol of our research group has been published previously [28]. High-resolution cine images on the long- and short axis covering the LV (10–12 slices) were acquired using a balanced steady state free precession (bSSFP) sequence with retrospective electrocardiographic (ECG) gating [29].

Standard software (Circle Cardiovascular Imaging, Calgary, AB, Canada) was used for post-processing analyses with the semi-automatic detection of LV endo- and epicardial borders [30]. Papillary muscles were excluded from the LV myocardial mass (LVMM) and were included in the LV volume.

An ECG-triggered, phase-sensitive inversion recovery sequence was used to obtain late gadolinium enhancement (LGE) images 15–20 min after the application of 0.2 mmol/kg of Gd-DO3A-butriol (Gadovist^®^, Bayer Vital GmbH, Leverkusen, Germany), with short-axis slices covering the entire LV [29]. A picture archiving and communication system (PACS) workstation (IMPAX^®^, Agfa HealthCare, Bonn, Germany) was used for IS quantification, whereas “hyperenhancement” was defined as +5 standard deviations above the signal intensity of remote LV myocardium [31,32]. IS was depicted as the percentage of total LVMM. Microvascular obstruction (MVO) was defined as a persisting area of “hypoenhancement” within the hyperenhanced territory and was also reported as a percentage of LVMM [31]. MVO regions were included in the aggregate IS.

Experienced observers who were blinded to clinical and angiographic data analyzed all of the CMR images.

### 2.3. Statistical Analyses

SPSS Statistics 27.0.1 (IBM, Armonk, NY, USA) and MedCalc v19.0.7 (Ostend, Belgium) were used for the statistical analyses. Continuous data are depicted as median with interquartile range (IQR), and categorical variables are expressed as numbers with corresponding percentages. The differences in the continuous and categorical variables between two groups were assessed by the Mann–Whitney U-test and Chi-square test, respectively. Correlations between continuous variables were tested with Spearman’s rank test. For multivariable testing, linear and binary logistic regression analyses were used to reveal the independent associated markers of LVEF and LVEF ≤ 40%, respectively. Parameters indicating significant association (*p* < 0.05) with LVEF and LVEF ≤ 40%, respectively, in univariable analysis were inserted into the multivariable model. There were no missing values. Z-scores were calculated to present odds ratios (OR) per 1 standard deviation increase. Receiver operating characteristic (ROC) curve analysis was performed to depict the area under the curve (AUC) for the prediction of LVEF ≤ 40%. Comparisons of the ROC curves were conducted according to DeLong et al. [33]. AUC values were classified as negligible (≤0.55), small (0.56–0.63), moderate (0.64–0.70), and strong (≥0.71), following Rice and Harris [34]. For all of the statistical calculations, a two-tailed *p*-value of <0.05 was defined as significant.

## 3. Results

### 3.1. Baseline Patient Characteristics

A total of 432 STEMI patients were included in this analysis. Baseline characteristics of the overall cohort (*n* = 432) as well as separately for patients with LVEF > 40% (*n* = 335, 78%) and LVEF ≤ 40% (*n* = 97, 22%) at CMR are depicted in Table 1. The median age of the overall cohort was 57 (IQR 51–65) years. LVEF ≤ 40% (22% of patients) was associated with advanced age (*p* = 0.010) and smoking (*p* = 0.002). Total ischemia time was 178 (IQR 120–262) min and did not differ between patients with LVEF ≤ 40% and> 40% (*p* = 0.407). Patients with LVEF ≤ 40% had anterior infarcts more often (*p* < 0.001) as well as lower TIMI flows pre (*p* = 0.018) and post-PCI (*p* = 0.006). No patient had symptomatic heart failure before STEMI. Patients with LVEF ≤ 40% had Killip class II more often (*p* < 0.001).

**Figure 1:**
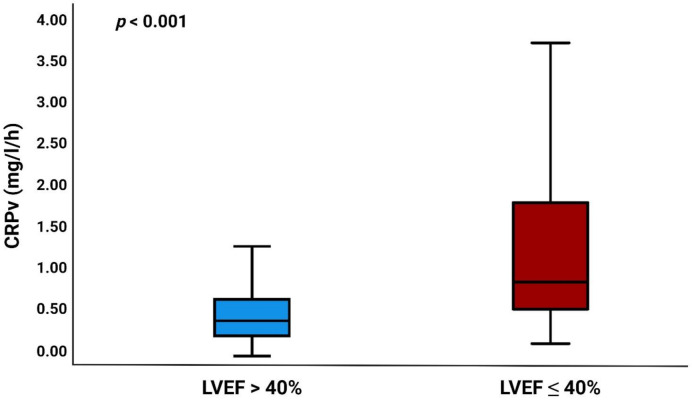
Boxplot showing the relation between CRPv and LVEF. CRPv = C-reactive protein velocity, LVEF = left ventricular ejection fraction.

The values for the median admission CRP, 24 h, and peak CRP were as follows: 2.1 (IQR 1.0–4.2), 12.4 (IQR 6.9–20.1), and 22.5 (IQR 11.7–45.5) mg/L, respectively. The median CRPv was 0.42 (IQR 0.21–0.76) mg/L/h and was significantly higher in patients with LVEF ≤ 40% (*p* < 0.001) (Figure 1).

The median time from PCI to CMR was 3 (IQR 2–4) days. Table 2 provides the CMR parameters of the overall cohort and according to the dichotomized LVEF at 40%.

### 3.2. CRPv as a Marker of LV Dysfunction

CRPv was correlated with LVEF (r_S_ = −0.397, *p* < 0.001). In multiple linear regression analysis, CRPv (β: −0.161, *p* = 0.004), peak cTnT (β: −0.343, *p* < 0.001), TIMI flow pre-PCI (β: 0.085, *p* = 0.045), TIMI flow post-PCI (β: 0.105, *p* = 0.010), and current smoking (β: 0.104, *p* = 0.015) were significantly related to LVEF (Table 3). After binary logistic regression analysis, CRPv (OR 1.71, 95% confidence interval (CI) 1.19–2.45; *p* = 0.004) and peak cTnT (OR 2.09, 95% CI 1.54–2.85; *p* < 0.001) remained independently associated with LVEF ≤ 40% (Table 4). In ROC analysis, 24 h CRP (AUC 0.73, 95% CI 0.69–0.77; *p* < 0.001), CRPv (AUC 0.77, 95% CI 0.72–0.81; *p* < 0.001), peak CRP (AUC 0.77, 95% CI 0.73–0.81; *p* < 0.001), and peak cTnT (AUC 0.77, 95% CI 0.73–0.81; *p* < 0.001) emerged as strong predictors of LVEF ≤ 40%. The best cut-off value of CRPv in predicting LVEF ≤ 40% was >0.59 mg/l/h, with a sensitivity of 70% and a specificity of 75%. According to C-statistics, the AUCs of CRPv and peak CRP (AUC difference: <0.01, *p* = 0.807) and CRPv and peak cTnT (AUC difference: <0.01, *p* = 0.784) did not differ. The combination of peak cTnT and CRPv (AUC: 0.81, 95% CI 0.77–0.85, *p* < 0.001) resulted in a higher AUC than peak cTnT alone for the prediction of LVEF ≤ 40% (AUC difference: 0.04, *p* = 0.009) (Table 5). The statistical significance of the calibration performance according to the Hosmer–Lemeshow test of the combination of CRPv and TnT was *p* = 0.063. Internal validity was assessed in 1000 bootstrap samples to estimate the optimism-corrected confidence intervals of the AUC of the combination of CRPv and TnT (BCa 95% CI 0.76–0.86, *p* < 0.001).

## 4. Discussion

The present study investigated the association of CRPv with LV dysfunction as assessed by CMR in patients with acute STEMI treated with primary PCI for the first time. The major findings can be summarized as follows: (a) Patients with elevated CRPv levels had significantly lower LVEF. (b) In the first week following acute STEMI, the association of CRPv and LV dysfunction remained significant after adjustment for clinical (peak CRP, peak cTnT, smoking, age, Killip class) and angiographical parameters (anterior infarct localization, TIMI flow pre- and post-PCI). (c) The predictive value of CRPv for LVEF ≤ 40% was strong and additive to peak cTnT. Taken together, these data indicate that CRPv represents a sensitive risk stratification tool in daily clinical practice, that is available in the very early phase after STEMI. Moreover, further studies could explore whether patients with increased CRP levels could benefit from individualized therapeutic strategies targeting the post-STEMI inflammatory response.

Among several inflammatory markers in the setting of myocardial infarction, CRP represents the most intensively explored marker. As an acute phase protein, CRP is released by hepatocytes after the stimulation of cytokines, primarily interleukin-6, about 6 h after the beginning of ischemic injury and peaks at day 2–3 thereafter [6,12]. Interleukin-6 is considered to increase the risk of adverse events after an acute coronary syndrome [35]. Furthermore, existing evidence shows that ischemic cell damage by CRP is complement dependent [36]. Increased CRP levels are associated with a greater extent of myocardial tissue damage [6,7,8,18], more severe LV dysfunction [18,37], and the occurrence of adverse events [9,10,11] after myocardial infarction. Furthermore, persisting inflammatory response in the chronic phase after STEMI can contribute to adverse LV remodeling [38]. CRP might therefore serve as an early biomarker for risk stratification after infarction.

Changes in CRP concentrations during myocardial infarction are considered to play a crucial role regarding adverse cardiovascular events, including death [13] and LV dysfunction, even years later [14]. In a study by Świątkiewicz et al. investigating 204 patients with first STEMI, elevated serial CRP during STEMI was associated with an increased risk of LV systolic dysfunction and heart failure [16]. Furthermore, elevated CRP values are also suggested to predict LV remodeling in this patient population [17].

Dynamics in inflammatory processes during myocardial infarction, as measured by CRPv, have recently been shown to predict microvascular pathology [19], which is a major prognostic determinant after STEMI [39]. In line with this, another study indicated that CRPv might be associated with short-term mortality after STEMI [21]. Moreover, CRPv is not only associated with a risk for adverse outcomes after STEMI, but is also related to the onset of new atrial fibrillation [20]. Atrial fibrillation is known to predict adverse outcomes after STEMI [40]. Furthermore, Zahler et al. revealed an association between CRPv and acute kidney injury after STEMI [22]. In the present study, we could corroborate and expand previous findings by showing that CRPv is strongly and independently associated with LV dysfunction after acute STEMI. In particular, this study may have clinical and research implications: firstly, CRPv emerged as an early and sensitive parameter for the prediction of LV dysfunction, as measured by CMR-assessed LVEF, improving individual risk assessment in this patient population at a very early stage. Secondly, as elevated CRPv levels are indicative for reduced LVEF in this study, CRPv may help to identify patients who might benefit from an anti-inflammatory and more extensive cardio protective treatment [41]. This hypothesis needs to be addressed in further studies.

Another important research question is whether CRP is only an associate or a mechanistic (causal) driver of LV dysfunction after STEMI. Indeed, the modulation of inflammatory processes have recently moved more and more into focus in the treatment of STEMI. The recently published CAMI-1 study [18] revealed that the CRP gradient was correlated with a greater extent of myocardial IS and reduced LVEF. By lowering CRP concentrations with CRP apheresis, the authors concluded that the correlation between CRP and myocardial IS and LV dysfunction was no longer detectable. The promising role of selective CRP apheresis in this setting needs further evaluation. The currently ongoing, prospective, randomized controlled “CRP Apheresis in STEMI” trial (ClinicalTrials.gov Identifier: NCT04939805) is investigating the effect of selective CRP apheresis on IS after acute STEMI and will provide important insights [42]. Moreover, the ASSAIL-MI trial [43] revealed that the intraprocedural administration of the interleukin-6 inhibitor Tocilizumab led to significant CRP reduction and consequently to an increased myocardial salvage, as assessed by CMR. Nevertheless, there was no difference in LVEF and IS between the experimental and control group. In experimental models, NLRP3 (NOD-like receptor family, pyrin domain-containing 3) inflammasome-targeted strategies might be beneficial in acute myocardial infarction [44]. In a mouse model of ischemia-reperfusion injury, the inhibition of NLRP3 inflammasomes has been shown to preserve myocardial function [45]. Another anti-inflammatory therapeutic strategy might be interleukin-1 blockade with anakinra, which has been suggested to potentially prevent heart failure after acute myocardial infarction [46]. Canakinumab, an interleukin-1b inhibitor, has been considered to have a dose-dependent reduction in the occurrence of heart failure in patients with prior myocardial infarction and elevated CRP [47]. However, research in this field is warranted to point out possible future directives in anti-inflammatory therapies after myocardial infarction.

To summarize, CRPv could help in the characterization of the dynamic inflammatory mechanism in the setting of acute STEMI as a time-dependent parameter and has important implications on myocardial infarct characteristics and outcome [19], as well as on remnant LV function before or at hospital discharge upon STEMI.

### Limitations

In this study, only stable STEMI patients with Killip class < 3 and a delay < 12 h were included [19]. The majority of STEMI patients present with Killip class < 3 [48]. However, the association of CRPv and LVEF might thus not be applicable to unstable patients, to late presenters, and to NSTEMI. Moreover, the results of this analysis might not be applicable to patients with symptomatic heart failure before STEMI. The TIMI myocardial perfusion grade was not systematically assessed in this cohort, although it might be a better discriminator than TIMI flow post PCI for poor prognosis after STEMI [49,50]. Furthermore, our scientific explanations are not transmissive to patients with an increased admission CRP value (above 15 mg/L), which are, however, a very small minority of patients (<4%) [19]. Finally, this study investigated the impact of CRPv on early LV dysfunction; thus, the results might not be transmissive to patients with LV dysfunction occurring in the chronic phase after STEMI. Further validation and research is needed to describe the exact role and significance of CRPv in this setting.

## 5. Conclusions

CRPv is independently associated with LV dysfunction, as determined by CMR, before or at hospital discharge in patients with acute STEMI treated with primary PCI. CRPv might help to identify patients who are at an increased risk for LV dysfunction at a very early stage after STEMI.

## Figures and Tables

**Table 1:** Baseline patient characteristics.

	Total Population (*n* = 432)	LVEF > 40% (*n* = 335, 78%)	LVEF ≤ 40% (*n* = 97, 22%)	*p*-Value
Age, years	57 [51–65]	56 [50–64]	58 [53–69]	0.010
Female, *n* (%)	81(19)	68 (20)	13 (13)	0.125
Body mass index, kg/m^2^	26.1 [24.4–28.7]	26.0 [24.4–28.7]	26.2 [24.7–28.7]	0.622
Current smoker, *n* (%)	247 (57)	205 (61)	42 (43)	0.002
Hyperlipidemia, *n* (%)	230 (53)	176 (53)	54 (56)	0.586
Diabetes mellitus, *n* (%)	35 (8)	25 (8)	10 (10)	0.366
Family history, *n* (%)	135 (31)	112 (33)	23 (24)	0.160
Hypertension, *n* (%)	191 (44)	148 (44)	43 (44)	0.979
Systolic blood pressure, mmHg	137 [117–154]	136 [117–154]	137 [118–152]	0.827
Diastolic blood pressure, mmHg	82 [72–95]	80 [72–94]	85 [76–100]	0.039
Symptomatic heart failure before STEMI, *n* (%)	0 (0)	0 (0)	0 (0)	-
Killip class, *n* (%)				<0.001
I	296 (69)	247 (74)	49 (50)	
II	136 (32)	88 (26)	48 (50)	
Total ischemia time, min	178 [120–262]	171 [120–260]	188 [129–267]	0.407
Culprit lesion, *n* (%)				<0.001
RCA	183 (42)	165 (49)	18 (19)	
LAD	189 (44)	124 (37)	65 (67)	
LCX	57 (13)	45 (13)	12 (12)	
RI	3 (1)	1 (1)	2 (2)	
Anterior infarction, *n* (%)	190 (44)	126 (38)	64 (66)	<0.001
Number of affected vessels, *n* (%)				0.656
1	260 (60)	201 (60)	59 (61)	
2	119 (28)	95 (28)	24 (25)	
3	53 (12)	39 (12)	14 (14)	
TIMI flow pre-PCI, *n* (%)				0.018
0	273 (63)	200 (60)	73 (75)	
1	55 (13)	43 (13)	12 (13)	
2	75 (17)	67 (20)	8 (8)	
3	29 (7)	25 (7)	4 (4)	
TIMI flow post-PCI, *n* (%)				0.006
0	4 (1)	2 (1)	2 (2)	
1	6 (1)	3 (1)	3 (3)	
2	34 (8)	20 (6)	14 (14)	
3	388 (90)	310 (92)	78 (81)	
CRP, mg/L				
Admission	2.1 [1.0–4.2]	2.0 [1.0–4.2]	2.3 [1.0–4.7]	0.383
24 h	12.4 [6.9–20.1]	11.0 [6.0–17.1]	20.9 [10.9–45.7]	<0.001
Peak	22.5 [11.7–45.5]	19.0 [10.3–34.4]	54.6 [25.9–94.7]	<0.001
Admission to 24 h CRP, h	21 [19–25]	21 [19–25]	21 [19–25]	0.640
Admission to peak CRP, h	46 [35–56]	45 [31–55]	47 [42–58]	0.028
CRPv (admission to 24 h), mg/L/h	0.42 [0.21–0.76]	0.34 [0.16–0.61]	0.81 [0.47–1.78]	<0.001
cTnT, ng/L				
Peak	4646 [2187–8430]	3902 [1718–6676]	9065 [5014–14877]	<0.001
Admission to peak cTnT, h	11 [7–16]	11 [7–16]	9 [6–13]	0.014

CRP = C-reactive protein, CRPv = C-reactive protein velocity, cTnT = cardiac troponin T, LAD = left anterior descending artery, LCX = left circumflex artery, LVEF = left ventricular ejection fraction, PCI = percutaneous coronary intervention, RCA = right coronary artery, RI = ramus intermedius, TIMI = thrombolysis in myocardial infarction.

**Table 2:** CMR imaging results.

	Total Population (*n* = 432)	LVEF > 40% (*n* = 335, 78%)	LVEF ≤ 40% (*n* = 97, 22%)	*p*-Value
LVEDV, mL	167 [137–189]	162 [134–187]	182 [154–204]	<0.001
LVESV, mL	83 [64–94]	75 [60–92]	118 [99–131]	<0.001
LVEF, %	49 [42–55]	-	-	-
LVSV, mL	79 [65–94]	84 [70–97]	60 [50–75]	<0.001
CO, L/min	5.3 [4.4–6.2]	5.5 [4.7–6.3]	4.6 [3.8–5.7]	<0.001
IS, % of LVMM	14.5 [7.5–24.3]	13.0 [6.2–20.6]	26.1 [16.0–34.2]	<0.001
MVO, *n* (%)	241 (56)	160 (48)	81 (84)	<0.001
MVO, % of LVMM	0.4 [0.0–2.5]	0.0 [0.0–1.5]	2.5 [0.6–6.4]	<0.001

CMR = cardiac magnetic resonance, CO = cardiac output, IS = infarct size, LVEDV = left ventricular end-diastolic volume, LVESV = left ventricular end-systolic volume, LVEF = left ventricular ejection fraction, LVMM = left ventricular myocardial mass, LVSV = left ventricular stroke volume, MVO = microvascular obstruction.

**Table 3:** Linear regression analysis for the prediction of LVEF.

	Univariable		Multivariable	
	**β**	***p*-Value**	**β**	***p*-Value**
CRPv	−0.397	<0.001	−0.161	0.004
Peak CRP	−0.378	<0.001	−0.098	0.082
Peak cTnT	−0.498	<0.001	−0.343	<0.001
Anterior infarction	−0.222	<0.001	−0.047	0.253
TIMI flow pre-PCI	0.264	<0.001	0.085	0.045
TIMI flow post-PCI	0.204	<0.001	0.105	0.010
Current smoker	0.173	<0.001	0.104	0.015
Age	−0.133	0.006	0.011	0.806
Diastolic blood pressure	0.000	0.999	-	-
Killip class	−0.210	<0.001	−0.053	0.197

CRP = C-reactive protein, CRPv = C-reactive protein velocity, cTnT = cardiac troponin T, LVEF = left ventricular ejection fraction, PCI = percutaneous coronary intervention. TIMI = thrombolysis in myocardial infarction.

**Table 4:** Binary logistic regression analysis for the prediction of LVEF ≤ 40%.

	Univariable		Multivariable	
	OR (95% CI)	*p*-Value	OR (95% CI)	*p*-Value
CRPv	2.69 (2.01–3.60)	<0.001	1.71(1.19–2.45)	0.004
Peak CRP	2.55 (1.92–3.39)	<0.001	1.28 (0.92–1.79)	0.146
Peak cTnT	2.82 (2.15–3.71)	<0.001	2.09 (1.54–2.85)	<0.001
Anterior infarction	1.78 (1.41–2.26)	<0.001	1.28 (0.97–1.71)	0.079
TIMI flow pre-PCI	0.67 (0.51–0.88)	0.003	1.04 (0.75–1.44)	0.828
TIMI flow post-PCI	0.73 (0.60–0.89)	0.002	0.83 (0.65–1.05)	0.112
Current smoker	0.70 (0.56–0.88)	0.002	0.76 (0.56–1.03)	0.079
Age	1.38 (1.09–1.72)	0.006	1.03 (0.75–1.37)	0.914
Diastolic blood pressure	1.27 (1.02–1.59)	0.037	1.18 (0.89–1.55)	0.258
Killip class	2.75 (1.72–4.38)	<0.001	1.54 (0.87–2.76)	0.142

CI = confidence interval, CRP = C-reactive protein, CRPv = C-reactive protein velocity, cTnT = cardiac troponin T, LVEF = left ventricular ejection fraction, OR = odds ratio, PCI = percutaneous coronary intervention. TIMI = thrombolysis in myocardial infarction. OR are presented per 1 standard deviation increase.

**Table 5:** C-statistics for the prediction of LVEF ≤ 40%.

Variables	AUC	95% CI	*p*-Value	AUC Increment	ROC Comparison
Admission CRP	0.53	0.48–0.58	0.383	-	-
24 h CRP	0.73	0.69–0.77	<0.001	0.20	<0.001
CRPv	0.77	0.72–0.81	<0.001	0.04	<0.001
Peak CRP	0.77	0.73–0.81	<0.001	<0.01	0.807
Peak cTnT	0.77	0.73–0.81	<0.001	<0.01	0.905
CRPv and peak cTnT	0.81	0.77–0.85	<0.001	0.04	0.009

AUC = area under the curve, CI = confidence interval, CRP = C-reactive protein, cTnT = cardiac troponin T, LVEF = left ventricular ejection fraction, ROC = receiver operating characteristic.
